# Mature Cystic Teratoma in Douglas' Pouch

**DOI:** 10.1155/2015/202853

**Published:** 2015-04-28

**Authors:** Kenji Ohshima, Anna Umeda, Ayako Hosoi, Toshiya Yamamoto, Satoru Munakata

**Affiliations:** ^1^Department of Pathology, Sakai City Hospital, 1-1-1 Minami-Yasui-Cho, Sakai-Ku, Sakai, Osaka 590-0064, Japan; ^2^Department of Obstetrics and Gynecology, Sakai City Hospital, 1-1-1 Minami-Yasui-Cho, Sakai-Ku, Sakai, Osaka 590-0064, Japan

## Abstract

Mature cystic teratoma is one of the most common ovarian neoplasms, but extragonadal teratoma is rare. Teratoma in Douglas' pouch is extremely rare, and only 12 cases have been reported since the first case was described in 1978. We report a 20-year-old woman with a multicystic mass in Douglas' pouch that was treated via laparoscopic resection. The tumor consisted of cysts lined by stratified squamous epithelium with an accumulation of keratin debris and various mature tissues. No immature elements or malignancy was found in the tumor, confirming the pathologic diagnosis of a mature cystic teratoma. The teratoma contained no ovarian tissues and both of the ovaries were intact on laparoscopy. These findings suggest that the teratoma originated primarily in Douglas' pouch rather than being caused by autoamputation of a previously existing ovarian teratoma. This is the first case that simultaneously showed normal ovaries and a teratoma in Douglas' pouch on laparoscopy.

## 1. Introduction

Mature cystic teratoma is the most commonly encountered ovarian neoplasm. However, teratomas in Douglas' pouch are extremely rare. Only 12 cases have been reported since Lefkowitch et al. described the first case in 1978 [1–5]. In most of these cases, the causes of the teratomas were thought to be autoamputation resulting from torsion with subsequent reimplantation in Douglas' pouch, because, in these cases, one of the ovaries was small or absent, or the teratomas contained ovarian tissues [2–5]. Other hypotheses have been proposed, such as teratomas in Douglas' pouch that may originate from an ectopic ovary or from displaced primordial germ cells. In this report, we present a case of teratoma in Douglas' pouch and discuss the etiology of the teratoma.

## 2. Case Presentation

**Figure 1:**
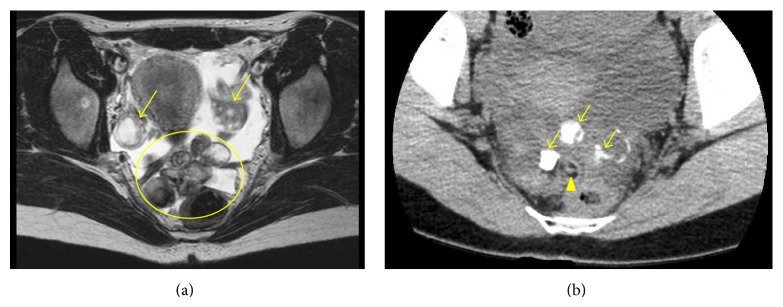
(a) T2 weighted magnetic resonance imaging showing a multicystic mass (circle) and normal ovaries (arrows). (b) Computed tomography showing calcified (arrows) and fatty (arrow head) components in the mass.

**Figure 2:**
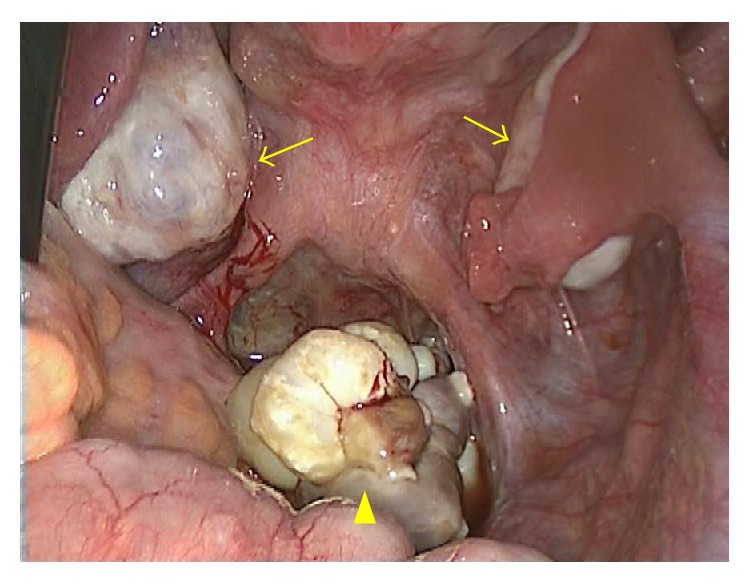
Laparoscopy showing normal ovaries (arrows) and a multilobular tumor in Douglas' pouch (arrow head).

**Figure 3:**
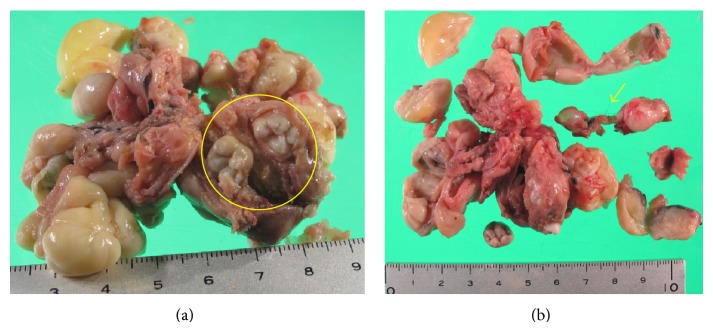
(a) Resected tumor (7.0 × 6.5 × 2.5 cm) and (b) cut surfaces. The tumor was multilobular and contained teeth, gingiva (circle), and hair (arrow).

**Figure 4:**
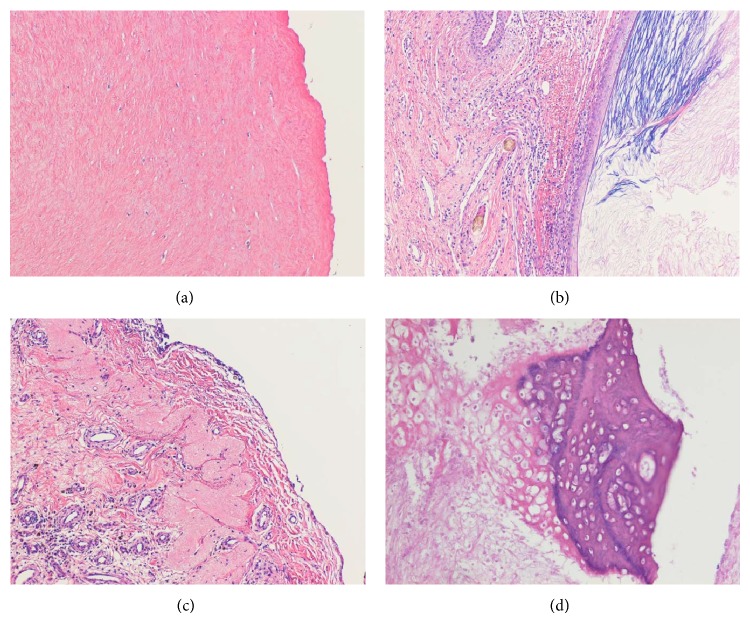
Microscopic findings of the tumor: (a) the tumor showed hyalinization. The main components of the tumor were (b) cysts and hair, (c) neural tissue, and (d) cartilage. (Hematoxylin and Eosin staining, original magnification ×4 (a, b, c), ×10 (d)).

A 20-year-old woman without a medical history presented with lower abdominal pain that had persisted for two weeks. A general physical examination was unremarkable except for tenderness of the lower abdomen. Transvaginal ultrasonography showed swelling of both of the ovaries, with the right ovary measuring 8 cm and the left, which was solid and multicystic, measuring 6 cm. Right ovarian hemorrhage and a left ovarian tumor were suspected. Because her vital signs were stable and the pain was mild, she was followed as an outpatient. A follow-up ultrasonography examination revealed that while both of the ovaries had decreased in size, the left ovary still measured 4 cm. The patient underwent a more detailed examination and we performed magnetic resonance imaging (MRI). MRI revealed a multicystic mass in Douglas' pouch while both of the ovaries were normal in size. The levels of tumor markers (CA-125, CA-19-9, alpha-fetoprotein, and carcinoembryonic antigen) were all normal. Subsequent contrast enhanced computed tomography (CT) showed fatty and calcified components in the mass, and the diagnosis of a teratoma was mainly considered (Figures 1(a) and 1(b)). Both MRI and CT scans showed that both of the ovaries were normal. Laparoscopic resection was performed, revealing a multilobular tumor in Douglas' pouch, free from surrounding tissues with a vascular pedicle arising from the retroperitoneum. Both of the ovaries were normal considering the patient's age (Figure 2). The tumor was resected without rupture. Macroscopically, the tumor was whitish, multilobular, and solid. The size of the tumor was 7.0 × 6.5 × 2.5 cm, and the tumor contained teeth, gingiva, and hair (Figure 3). On microscopic examination, the tumor showed remarkable hyalinization (Figure 4(a)). It consisted of cysts lined by stratified squamous epithelium with an accumulation of keratin debris and various mature tissues such as hair, teeth, cartilage, neural tissue, fat, foreign body granuloma, and granulation tissue (Figures 4(b)–4(d)). The tumor contained no ovarian tissues. No immature elements or malignancy was found in the tumor, confirming the final pathologic diagnosis of mature cystic teratoma. The patient had an uneventful postoperative course.

## 3. Discussion

Mature cystic teratoma is one of the most common ovarian neoplasms, accounting for approximately 20% of all adult ovarian neoplasms [6]. Teratomas may occur at extragonadal sites, usually along the midline of the body such as the mediastinum. However, extragonadal teratomas in the abdominal cavity are extremely rare, and the most commonly reported cases are in the omentum, 32 cases in total [7, 8]. Only 12 cases of teratomas in Douglas' pouch have been reported to date [1–5]. This is the first case that simultaneously showed normal ovaries and a teratoma in Douglas' pouch on laparoscopy.

The symptoms observed in this patient were abdominal pain with bilateral ovarian enlargement on transvaginal ultrasonography. The right ovary decreased in size but the left sustained a size up to 4 cm on ultrasonography. The patient was not receiving any medication and her menstrual cycle was regular, so the possibilities of ovarian hyperstimulation syndrome or polycystic ovary syndrome were excluded. We thought that right ovarian hemorrhage had caused the abdominal pain and that a left ovarian tumor had been found incidentally. However, MRI, CT, and laparoscopy revealed normal ovaries and a multicystic mass in Douglas' pouch. This most likely indicated that the left ovarian tumor we had first recognized on transvaginal ultrasonography was actually the tumor in Douglas' pouch.

Differential diagnoses of a cystic mass in Douglas' pouch include cystic mesothelioma, cystic lymphangioma, and endometriotic cyst. It is difficult to establish a diagnosis of teratoma in Douglas' pouch preoperatively. The preoperative diagnostic indication of teratoma is the presence of fat and calcification on CT images. Other differential diagnoses of a tumor with multifocal calcification and fat tissue components of Douglas' pouch are malignant peritoneal mesothelioma, liposarcoma, and metastasis of carcinoma. In our case, we observed the presence of fat and calcified components on the CT images. We considered the diagnosis of teratoma first, taking into account the patient's age, sex, and laboratory data. The tumor was successfully treated via laparoscopic resection, and pathological diagnosis of mature cystic teratoma was finally made. Pathological examination is important. Immature teratoma or teratoma with malignant transformation must be differentiated.

The etiology of extragonadal teratomas is poorly understood but is thought to arise from primordial germ cells or early embryonic cells or from totipotent cells [9]. Three main theories have been proposed to explain the etiology of extragonadal teratomas. The first theory is that extragonadal teratomas may be autoamputated into the abdominal cavity. When one of the ovaries is small or absent, or teratomas contain ovarian tissues, autoamputation has been thought to be a possible cause. This theory is widely accepted as the etiology of extragonadal teratomas in the abdominal cavity and 6 cases of teratomas in Douglas' pouch have been thought to be due to autoamputation [2–5]. However, in our case, the teratoma contained no ovarian tissues and both of the ovaries were intact on laparoscopy. Thus, autoamputation may not be a reasonable theory in our case. The second theory is that extragonadal teratomas may occur in an ectopic ovary [5]. An ectopic ovary is thought to arise congenitally or following a surgical procedure or inflammation within the pelvis. Our patient had no history of laparotomy, and the tumor contained no ovarian tissues. Therefore, this theory is not applicable. The third theory is that extragonadal teratomas may originate from displaced primordial germ cells. During fetal development, primordial germ cells migrate from the yolk sac along the hindgut, to the midline of the body, towards the genital ridge. Primordial germ cells may stop during migration and then may cause a teratoma later [9]. We think this theory is most applicable to our case, even though it is difficult to prove. During migration to the genital ridge, DNA methylation of primordial germ cells is erased and then reestablished during gametogenesis [10]. The results of genomic imprinting research have indicated that each group of germ cell tumors has a different imprinting status [11]. For example, teratoma/yolk sac tumors in infants and teratomas of the ovary in adults have a different status of imprinting. If we could have investigated the imprinting status of the teratoma in our case, it may have been helpful in understanding the origin of the tumor.

In conclusion, we report an extremely rare case of the teratoma in Douglas' pouch. Because the teratoma had no ovarian tissues and both of the ovaries were normal in size; the teratoma was considered to originate primarily in Douglas' pouch.
